# Hazardous Alcohol Consumption Depicted in YouTube Videos: A Content Analysis

**DOI:** 10.7759/cureus.108600

**Published:** 2026-05-10

**Authors:** Carissa N Bartkowiak, Larissa Dean, Dalton King, Emily Ulrich, Skyler Steward, Emma Lindemann, Michelle A Padley, Brian G Lewis, Bryan Judge, Jeffrey S Jones

**Affiliations:** 1 Emergency Medicine, Michigan State University College of Human Medicine, Grand Rapids, USA; 2 Emergency Medicine, Corewell Health Grand Rapids Hospitals - Butterworth, Grand Rapids, USA; 3 Toxicology, Corewell Health Grand Rapids Hospitals - Butterworth, Grand Rapids, USA; 4 Toxicology, Michigan State University College of Human Medicine, Grand Rapids, USA

**Keywords:** alcohol, content analysis, hazardous alcohol consumption, hazardous effects, social media, toxicology, youtube

## Abstract

Aims and objectives

Social media platforms such as YouTube allow adolescents and young adults to document substance use and share related beliefs with large audiences. This study primarily aimed to identify and describe hazardous alcohol consumption methods depicted in highly viewed YouTube videos. Secondary exploratory objectives were to characterize the apparent demographic characteristics of on-screen performers, quantify video popularity and engagement, and evaluate the quality, reliability, and scientific accuracy of alcohol-related information presented in these videos.

Methods

We conducted a retrospective content analysis of YouTube videos identified between January and March 2025 using 48 search terms related to hazardous alcohol use. Key variables included the number of views, the number and apparent characteristics of participants, and the method of alcohol consumption depicted. Video content and quality were assessed using the global quality score (GQS) and a modified DISCERN reliability score, and explicit scientific claims were classified as substantiated or unsubstantiated by two board‑certified toxicologists.

Results

A total of 278 videos involving 15 distinct methods of alcohol consumption were analyzed, most of which met predefined criteria for hazardous use. Risky practices included alcohol inhalation, alcohol enemas, vodka eyeballing, drunkorexia, funneling, drinking hand sanitizer, marijuana moonshine, and alcohol‑soaked tampons, among others. Only three videos (1.1%) contained trigger warnings. Collectively, the videos were viewed 75 million times (mean 269,784 views) and liked five million times, and they featured 722 participants or observers, predominantly male, Caucasian, and aged 21-25 years. The median GQS and reliability scores were 1 (interquartile range or IQR 2-3) and 1 (IQR 1-2), respectively, and 78.7% (159/202) of scientific claims in informational videos conflicted with published toxicology literature. Interrater agreement was substantial to excellent (Cohen’s kappa 0.66-0.76).

Conclusions

Hazardous alcohol use is highly visible in popular YouTube videos, which rarely include accurate risk information or explicit harm‑reduction messages. These low‑quality, often misleading depictions suggest that YouTube and similar platforms may contribute to alcohol‑related informational environment for the youth and serve as venues for future efforts to address misinformation and hazardous drinking norms.

## Introduction

Alcohol use remains a major public health concern among adolescents and young adults because it contributes substantially to injury, disability, and premature death worldwide [1,2]. For people aged 15 to 29 years, alcohol is a leading risk factor for morbidity and mortality, particularly when consumption involves binge drinking or other hazardous patterns of use [2-5]. These behaviors are associated with acute harms such as injury and poisoning as well as broader educational, social, and economic consequences.

At the same time, social media channels, such as YouTube, have become a central part of how young people encounter, interpret, and share health-related behaviors, including alcohol use [6-8]. Exposure to alcohol-related content online has been associated with greater alcohol use, more permissive attitudes toward drinking, and misperceptions of peer norms [6-9]. Platforms that emphasize visual and video content can make risky behaviors appear entertaining, socially rewarded, and easy to imitate while minimizing visible negative consequences. For adolescents who rely heavily on peers and influencers as information sources, such content can blur the boundary between entertainment, informal marketing, and health information [10].

YouTube is one of the largest video-sharing platforms and contains a vast amount of user-generated alcohol-related content, ranging from casual drinking to extreme or medically dangerous practices [11-13]. Prior research has examined alcohol imagery, intoxication portrayals, and online alcohol marketing on YouTube and other platforms, but comparatively few studies have focused on specific high‑risk consumption methods, the apparent characteristics of the individuals performing them on screen, and the reliability of the information conveyed in such videos [6-8]. Although other platforms, such as TikTok and Instagram Reels, have rapidly grown as venues for short-form video content, YouTube remains a widely used platform among adolescents and young adults. It's relatively stable, with longer-form video archives, a transparent search interface, and publicly available engagement metrics, making it well-suited for systematic content analysis. The substantial existing literature on alcohol and health-related YouTube content provides important context for comparison over time [11-13].

Importantly, the people who appear in these videos are not necessarily the same as the audiences who watch them. Many on-screen performers appear to be legal-age young adults, yet their videos remain readily accessible to adolescents and younger viewers, who may use this content to form expectations about what drinking looks like or what behaviors are socially acceptable. This distinction is important because the public health concern is not limited to who performs the behavior but also includes how hazardous alcohol use is portrayed and normalized in digital spaces frequented by youth.

Accordingly, this study primarily aimed to characterize hazardous alcohol consumption as it is depicted in highly viewed YouTube videos by identifying and describing the drinking behaviors shown. Secondary exploratory objectives were to characterize the apparent demographic characteristics of on-screen performers, quantify the popularity and engagement of these videos, and evaluate the quality, reliability, and scientific accuracy of the alcohol-related information and claims they present. By focusing on what is depicted and how it is framed, this study seeks to clarify the types of hazardous alcohol content that adolescents and young adults may encounter on YouTube and to identify opportunities for clinical counseling, harm-reduction messaging, and public health intervention.

## Materials and methods

Search strategies and procedures

We conducted a retrospective content analysis over three months in 2025 (January to March 2025). Using YouTube’s search engine, we identified videos using 48 specific terms relating to hazardous alcohol consumption. The complete list of search terms is provided in the Appendix. Search terms were generated from prior literature on hazardous drinking methods, informal scanning of YouTube content, and team brainstorming. Terms were then standardized by removing duplicates, consolidating closely related phrases, and fixing spelling and phrasing across searches. In this study, the term “alcohol” refers specifically to ethanol in beverages and ethanol-containing products intended for human consumption and does not include other alcohols such as methanol or isopropanol.

The inclusion criteria were English-language audio, a duration of at least 30 seconds, and alcohol-related content depicting hazardous or risky consumption methods. A recent study showed that only 6.6% of users are willing to go to the second page (or beyond) on a Google search [14]. As a result, only the videos that appeared on the first two pages for each keyword were evaluated.

**Figure 1:**
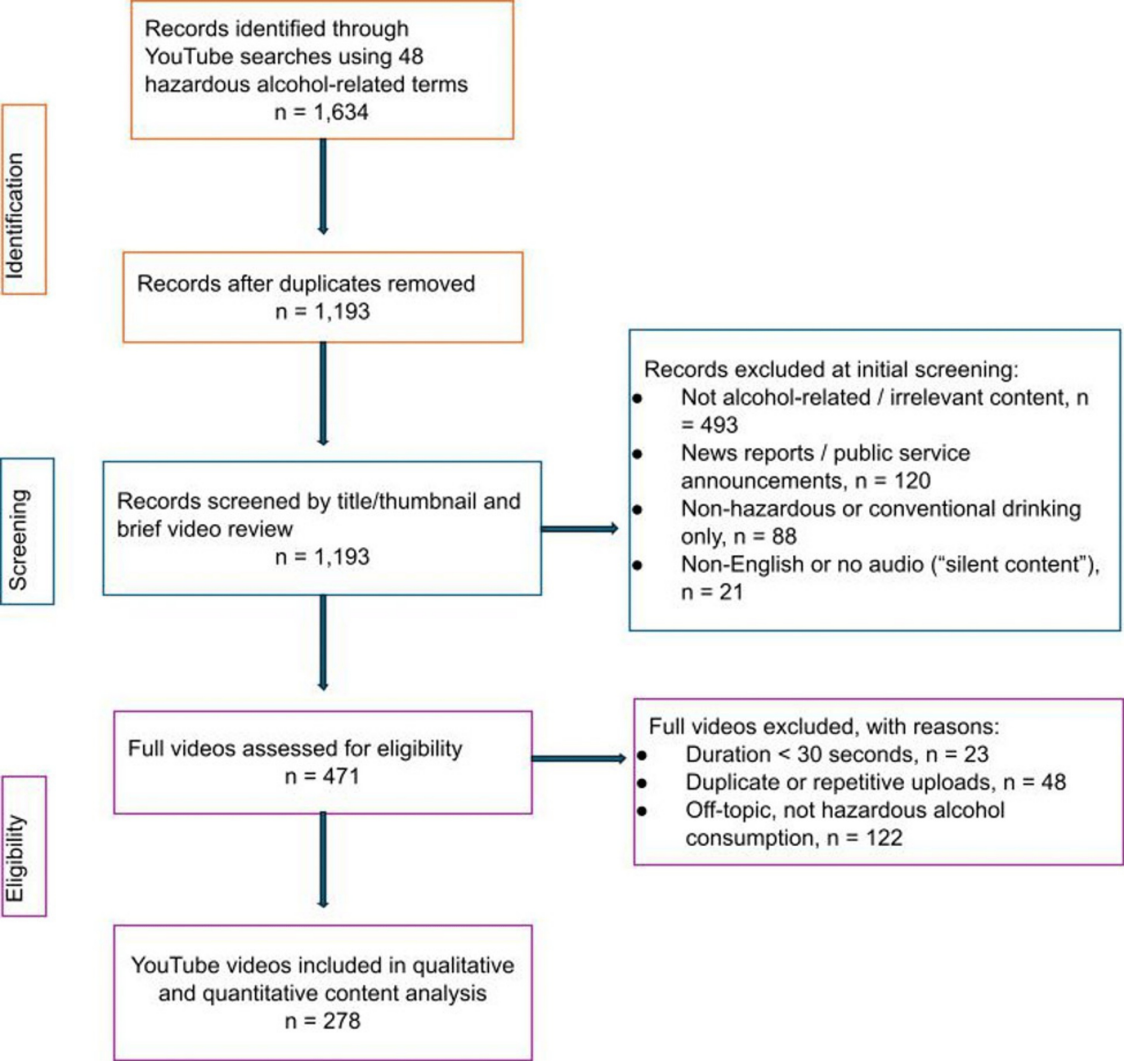
PRISMA-style flow diagram showing the identification, screening, eligibility assessment, exclusion, and final inclusion of YouTube videos in the content analysis PRISMA: Preferred Reporting Items for Systematic Reviews and Meta-Analyses.

Two trained student reviewers independently and in parallel screened all search results to determine eligibility, with uncertainties resolved in consultation with a senior author. Videos that were duplicate or repetitive uploads, contained irrelevant content, or were news reports or public service announcements were excluded. Figure 1 presents a flow diagram of video identification, screening, exclusions by reason, and the final sample of 278 included videos.

To reduce personalization and improve reproducibility, searches were conducted during the defined study period using a standardized browser configuration with no prior watch history and default YouTube search settings. Because YouTube search results are dynamic and may vary by time, location, and user-specific factors, URLs for all included videos were archived at the time of data collection to create a stable sampling frame for analysis. This study was exempt from ethical approval because of the observational design, with videos readily available to the public.

Principal data abstraction

Key quantitative and qualitative descriptive variables included the number of views, participants, accuracy of the information provided, and the method of alcohol consumption. View counts and “like” counts were recorded on the date each video was coded during the January-March 2025 data collection period, and all aggregate metrics reported reflect those values. For each video, coders also recorded the apparent age group, sex, and race/ethnicity of the on-screen participants based on visible appearance and contextual cues; these were estimates rather than verified demographic data.

Viewer comments were examined as an exploratory index of audience response. For each included video, the first 10 visible comments, sorted by YouTube’s default “Top comments” setting, were extracted and analyzed using a structured thematic approach to identify dominant patterns, such as praise or validation, sharing similar experiences, humor, risk minimization, or explicit concern about safety.

A single trained coder applied a predefined codebook to assign comments to thematic categories, with ambiguous cases discussed among investigators to ensure consistent interpretation. This component was considered exploratory and was not subjected to separate formal interrater reliability testing.

The content and quality of the videos were evaluated using the Global Quality Scale (GQS) and a modified DISCERN reliability score. The GQS is a five-point Likert-type instrument originally developed to assess the overall quality, flow, and usefulness of online health information for patients. In this study, the GQS was scored from one (low quality) to five (excellent quality), reflecting overall quality, information flow, and educational value, as described previously [15]. The reliability of consumer health information was assessed using a five-point modified DISCERN tool, adapted by Shepperd et al. [16] from the original DISCERN tool developed by Charnock et al. [17]. The modified DISCERN score comprises five yes/no items assessing whether the video had clear aims, used reliable sources, presented balanced, unbiased information, listed additional sources, and acknowledged areas of uncertainty. One point was assigned for each criterion met, yielding a total score from zero to five, with higher scores indicating greater reliability. This tool is commonly used for auditing YouTube and social media content, focusing on clarity, source reliability, balance, and uncertainty. The exact item set and scoring rubrics used in this study are provided in the appendices.

Misinformation and scientific claims

The scientific information and claims (e.g., explicit scientific or quasi-scientific statements about alcohol risks, mechanisms, or effects) in the videos were classified as substantiated or unsubstantiated by two board-certified toxicologists. Each distinct statement was coded separately, and multiple claims could occur within a single video. Both toxicologists participated in protocol training before the project began, along with the rest of the research team members, and independently reviewed the claims using predefined criteria. Unsubstantiated claims were identified using criteria shown in Table 1, including absence of supporting evidence, contradiction with scientific consensus, reliance on low-quality sources, lack of reproducibility, and ambiguity or exaggeration. 

**Table 1:** Review criteria for classifying unsubstantiated medical claims in video content [17,18]

Definition	Description
Absence of evidence	The claim is not supported by systematic reviews, randomized controlled trials, well-designed observational studies, or official statements from recognized health authorities.
Contradicted by scientific consensus	The statement is inconsistent with or directly contradicts by the current consensus in the relevant medical or scientific community.
Reliance on anecdote or low-quality sources	The claim is based solely on anecdotal reports, non-peer-reviewed sources, outdated information, or expert opinion without corroborating evidence.
Lack of reproducibility	The statement cannot be consistently verified across multiple, independent and high-quality sources.
Ambiguity or exaggeration	The claim is vague, misleading, or exaggerated beyond what available evidence supports.

Initial agreement on the 10% subsample was 93.3%, with a Kappa of 0.78. Discrepancies were resolved through discussion to generate the final consensus ratings used in analyses.

Training of YouTube content coders

At the outset of the study, project investigators met to review the data abstraction tools and coding scheme. Before project initiation, all the study team members completed protocol-specific training on study objectives and primary endpoints, inclusion and exclusion criteria for content screening, the data extraction approach, and the content analysis methodology. Six student reviewers were mentored by a senior author (JSJ), who provided expertise and guidance throughout the screening and extraction process. Each eligible video underwent structured data abstraction by one trained reviewer using a standardized coding form. Additionally, the senior author met regularly with reviewers to ensure consistency in abstraction and to resolve any questions that arose.

To assess interrater reliability, faculty physicians independently reviewed a random sample of 10% of included videos. Agreement statistics for key coding dimensions-hazardous alcohol method classification, GQS score, modified DISCERN score, and the presence or absence of misinformation-were calculated using Cohen’s kappa. These kappa values were derived from the subsample rather than the full dataset. Furthermore, to ensure quality control and reduce interobserver variation, an additional faculty member reviewed the collected data sheets. Any queries or concerns regarding content assessment were resolved during discussion, and there were no conflicts that required mediation by an additional author. Following data abstraction, responses were drafted into a spreadsheet format for thematic coding and analysis. All statistical analyses were conducted with SAS Statistical package v.9.4 (SAS Inc., Cary, NC, US).

Study endpoints

Primary outcomes of the study were: (1) the number of YouTube videos depicting hazardous alcohol consumption and (2) the hazardous drinking behaviors and methods of alcohol consumption depicted in these videos. Hazardous alcohol consumption methods in this study were defined as behaviors or routes of administration that substantially increase the risk of acute harm (e.g., injury, poisoning, aspiration, chemical or thermal injury, overdose, medical or psychiatric emergency, or longer-term health consequences) beyond those associated with conventional oral alcohol use at comparable doses. This two-tier classification, distinguishing hazardous routes from behaviorally high-risk patterns, was developed a priori by board-certified toxicology experts using predefined criteria and supporting literature, but does not represent a formally validated scale

Secondary (exploratory) outcomes included: (1) the popularity and engagement of these videos, measured by view and “like” counts; (2) the apparent demographic characteristics of on‑screen performers; and (3) the proportion and nature of videos that contained inaccurate or misleading medical or scientific information about alcohol. Descriptive statistics and frequency tables were used to summarize all outcomes.

## Results

During the study period, 278 YouTube videos were analyzed. These videos described 15 different methods of consuming alcohol. The more dangerous practices described were alcohol inhalation, alcohol enemas, vodka eyeballing, drinking hand sanitizer, marijuana moonshine, alcohol-soaked tampons, and inhaling alcohol powder through the nose. Eight behaviors were hazardous because they promote rapid or excessive consumption, social pressure to drink beyond personal limits, or engagement in risky dares or competitions, thereby increasing the likelihood of acute intoxication, injury, impaired judgment, or other adverse outcomes beyond typical social drinking (Table 2).

**Table 2:** Hazardous methods of alcohol consumption observed in YouTube videos ^a^Behaviors or routes of administration that substantially increase the risk of acute harm, medical or psychiatric emergency, or longer-term health consequences, beyond those associated with conventional oral alcohol use at comparable doses [1-5]. ^b^Alcohol use behaviors that promote rapid or excessive consumption, social pressure to drink beyond personal limits, or engagement in risky dares or competitions, thereby increasing the likelihood of acute intoxication, injury, impaired judgment, or other adverse outcomes beyond typical social drinking [3,5,7].

Clearly hazardous / medically dangerous routes or products^a^
Alcohol inhalation (Vapor, Vaportini, Smoking alcohol)
Alcohol enemas ((Butt chugging, Boofing, Booty bumping)
Eyeballing (Vodka eyeballing)
Drinking hand sanitizer
Cannabis/alcohol infusion (Marijuana moonshine)
Alcohol-soaked tampons (Slimming)
Alcohol powder (Snorting alcohol)
Hazardous binge/intoxication patterns or “challenges” (behaviorally high risk)^b^
Drunkorexia
Funneling (Beer bong)
Borg (Blackout rage gallon)
PK (Player-knockout)
Rainbow drink challenge (Drink the rainbow)
Drunken gummies (alcohol gummies)
Neknominate (Necking, Neknomination Or Neck nomination)
First drink, Last drink challenge

Only three of these videos (1.1%) posted trigger warnings intended to warn users that website content was inappropriate for some users.

As secondary (exploratory) outcomes, these videos were collectively viewed 75 million times on YouTube (mean 269,784 views per video). They were marked as “liked” a total of five million times (mean 17,985 likes per video), corresponding to an overall like‑to‑view ratio of 6.6%. Most videos explicitly encouraged or challenged viewers to participate in the drinking activity being demonstrated. Negative consequences and explicit risk messages were infrequently emphasized (21/278; 7.6%) and, when present, were typically subtle or secondary to the main entertainment focus. Most viewer comments validated and/or praised uploaders for their videos (1872/2702; 69.3%); in 9% (25/278) of cases, at least one comment explicitly raised safety concerns (Table 3).

**Table 3:** Types of viewer responses to the videos depicting hazardous alcohol consumption

Expressions of amusement, admiration, or encouragement (e.g., “this is hilarious,” “legend,” “I want to try this”).
Describing doing the same or very similar behaviors (e.g., “we used to do this in college,” “I’ve done this with my friends”).
Comments that highlight how much or how often they drink (e.g., “this is how I drink every weekend,” “I’d finish that whole bottle myself”).
Normalizing or glamorizing intoxication, attractiveness, and party identity, often with lots of likes and positive sentiment.
Responses indicating desire or intent to imitate (e.g., “doing this tonight,” “saving this for the weekend”).
Reflect “drinkers like me” identification and a spiral of positive feedback that reinforces risky norms.
Often social in nature, functioning to circulate alcohol content within peer networks.
Comments minimizing severity or framing the behavior as harmless fun (e.g., “everyone does this,” “it’s not that serious,” “lighten up”).
Comments that call out danger, health risks, or inappropriateness (e.g., “this is so dangerous,” “don’t do this at home”).

Many viewers shared their own alcohol experiences, while a few called out danger, health risks, or the inappropriateness of behavior.

In total, 722 participants or observers were identified in the videos. Based on appearance and contextual cues, most identified participants appeared to be male (527/722, 73.0%), Caucasian (496/722, 68.7%), and between 21 and 25 years of age (353/711, 48.9%), suggesting a relatively narrow apparent demographic profile. These predominantly young adult participants may function as perceived role models for some adolescent viewers; however, we lack viewer demographics or analytics to directly measure adolescent exposure, so this interpretation is speculative.

The median GQS was 2 (interquartile range or IQR 2-3), and the median modified DISCERN reliability score was 1 (IQR 1-2), indicating that most videos had low educational quality and very poor reliability overall [15-17]. Across 91 informational videos, we identified 202 discrete scientific claims (approximately 2.2 claims per informational video). Each claim was coded separately, and 159 of these 202 claims (78.7%) conflicted with published toxicology literature, indicating that nearly four out of five coded statements were inconsistent with current evidence (Table 4).

**Table 4:** Categories of alcohol-related misinformation in YouTube videos

Metabolism and toxicity claims
Statements that alcohol “leaves your system faster” with certain tricks (e.g., energy drinks, cold showers, specific foods).
Claims that non‑oral routes (inhalation, enemas, eyeballing) are “less toxic” because they “bypass the liver.”
Safety of alternative consumption methods
Asserting that inhaling, insufflating, or rectal use of alcohol is “safer” because it avoids calories or stomach irritation.
Reassurances that these methods are “just a different way to get the same buzz” without added risk.
Misrepresentation of “moderate” benefits
Overstating cardiometabolic or stress‑relief “benefits” of drinking without dose or context.
Presenting heavy episodic use as compatible with “moderate” drinking because it happens infrequently.
No safe level of alcohol consumption
Misusing slogans (e.g., “no safe level”) to justify nihilism (“if any amount is bad, might as well go hard”).
Equating any use with extreme harm in ways that distort actual dose–response data.
Detoxification myths
Promoting home “detox” hacks (supplements, special drinks) as ways to rapidly reverse intoxication or organ damage.
Suggesting that brief periods of abstinence fully “reset” the body regardless of prior heavy use.
Underestimation of social and physiological harms
Minimizing risks of aspiration, injury, or overdose (“you just puke and you’re fine”).
Framing blackouts, vomiting, or ED visits as funny or inconsequential outcomes.
Systemic misinformation
Presenting anecdote as equivalent to scientific evidence (“we’ve done this for years, so it’s safe”).
Rejecting expert or guideline-based information as exaggerated or “fear mongering.”
Absence of harm‑reduction context
Demonstrating hazardous behaviors without any mention of safer alternatives, dose limits, or when to seek care.
Failing to include age restrictions, content warnings, or advice against imitation.

Much of this misinformation involved inaccurate claims about alcohol metabolism and toxicity, unfounded assurances about the safety of alternative consumption methods, and misrepresentation of supposed “moderate” benefits or the idea that there is “no safe level” in ways that conflicted with current evidence. Additional themes included detoxification myths, consistent underestimation of social and physiological harms, broader systemic misinformation about alcohol‑related risk, and an almost complete absence of harm‑reduction context or practical safety advice. To assess interrater reliability, two reviewers independently coded a random 10% sample of videos. Cohen’s kappa values for this subset were 0.76 for hazardous alcohol method classification, 0.71 for GQS scores, 0.68 for modified DISCERN scores, and 0.66 for the presence or absence of misinformation. These values indicate substantial to excellent agreement across the primary coding dimensions.

## Discussion

This analysis of YouTube videos revealed that hazardous alcohol consumption methods were widely visible, highly engaging, and infrequently framed as dangerous. Videos depicting extreme or non‑traditional routes of alcohol administration, such as inhalation, enemas, eyeballing, drinking hand sanitizer, marijuana moonshine, alcohol‑soaked tampons, and intranasal alcohol powder, collectively attracted tens of millions of views and millions of “likes”.

Although YouTube does not publish formal benchmarks for typical video performance, available platform-wide analyses suggested that most videos receive relatively low view counts, often in the tens of views, and only a small minority exceed 10,000 views [19]. In contrast, the hazardous drinking videos in our sample collectively accrued 75 million views and five million likes, corresponding to a like‑to‑view ratio of roughly 6.6%, which is at or above commonly cited engagement benchmarks for YouTube content [20]. This study characterized hazardous alcohol consumption as depicted in highly viewed YouTube videos, focusing on the behaviors shown and the apparent demographics of on-screen performers, rather than on viewer characteristics. These metrics suggest that the extreme and nontraditional drinking behaviors we observed occupy a highly visible and highly engaging niche on the platform, although our study was not designed to determine their proportional significance within the broader ecosystem of alcohol-related YouTube content.

This level of popularity suggests that these behaviors are being strongly reinforced and normalized within online youth cultures [11-13]. At the same time, videos that promoted rapid or competitive oral consumption (e.g., funnels, “Borgs,” challenges) normalized heavy episodic drinking as a form of entertainment, often encouraging viewers to participate or emulate the behavior [6-9,11-13]. These findings support prior research, demonstrating that YouTube functions as an amplifier of risky alcohol norms among youth and young adults, with frequent exposure to alcohol‑related content associated with higher levels of use and more permissive attitudes [6-9].

The demographic profile of onscreen participants further highlights their potential to serve as behavioral models for viewers. Across 722 identified individuals, the majority were male, Caucasian, and between 21 and 25 years of age, reflecting a relatively narrow slice of drinkers who publicly stage and share risky behaviors. These predominantly young adult participants may serve as perceived role models for some adolescent viewers, consistent with social learning theory. However, we lacked viewer demographics or analytics to directly measure adolescent exposure, so this interpretation is speculative. Prior work suggests that repeated exposure to peer and influencer alcohol posts is associated with attitudes, perceived norms, misperceptions of peer behavior, and subsequent drinking among adolescents, especially when content is delivered by peers and influencers on social platforms, although these studies are largely observational and cannot establish causality [6-10].

Our findings on quality and reliability were especially troubling. The median GQS and modified DISCERN scores were low, indicating that most videos lacked clear educational goals, coherent structure, and trustworthy health information. This aligns with earlier studies of health-related YouTube content that identified similar issues across various clinical topics [21,22].

Among the videos that included explicit informational or scientific claims, nearly four out of five contradicted established toxicology literature, with frequent misinformation about metabolism, toxicity, safety of alternative routes, “detox” strategies, and the extent of social and physiological harms (Table 4).

The almost complete absence of explicit risk messages, combined with a systematic underestimation of harms, supports previous findings that alcohol-related YouTube content predominantly depicts drinking positively or neutrally and seldom provides strong warnings or harm-reduction advice [11-13]. Our findings suggest that many viewers are exposed to persuasive yet unreliable narratives that could undermine evidence-based counseling and public health messages about alcohol risks [6-9,13].

Despite these concerns, our findings highlight several potential intervention directions that warrant further study. Platforms that disseminate hazardous drinking trends may also be suitable venues for targeted counter-messaging, digital brief interventions, or youth-friendly harm-reduction content designed to correct misinformation and contextualize risk [6-9,11-13]. Clinicians may also consider asking about online alcohol-related content exposure, particularly when discussing hazardous drinking behaviors or misconceptions about safety and detox strategies, although the effectiveness of such approaches was not evaluated in this study. Public health practitioners and policymakers might also explore collaborations with content creators, clearer age-gating and warning labels, or platform strategies that surface accurate information alongside high-risk content [6-9,12-14].

This study has several limitations. First, our sampling relied on YouTube’s search engine and was restricted to the first two pages of results for each search term. Although this mirrors typical web-search behavior, it may have missed less searchable yet influential content, limiting generalizability beyond highly visible videos. Because our sampling relied on YouTube’s proprietary and evolving recommendation and ranking algorithms, our dataset likely overrepresents highly surfaced content and underrepresents less visible videos, further constraining generalizability and complicating reproducibility. All searches were conducted between January and March 2025, so our findings reflect a cross-sectional snapshot of content available at that time rather than a stable or exhaustive archive.

Second, we analyzed only English‑language videos of at least 30-second duration, so patterns of hazardous consumption and misinformation may differ in other languages, cultural settings, or short‑form formats such as YouTube Shorts or other platforms [6-9,11-13]. We did not systematically record whether videos were age-restricted, required sign‑in, or otherwise subject to YouTube age‑gating or content-moderation policies, so accessibility to unverified minors could not be determined.

Third, the demographic characteristics of the participants were inferred from visible appearance and contextual cues rather than verified data, introducing potential misclassification and limiting precision, particularly for race/ethnicity. Our classification of methods as hazardous is based on structured expert judgment rather than on a validated hazard rating instrument, so some misclassification is possible and may influence the reported number of ‘hazardous’ videos. 

Fourth, although GQS, modified DISCERN, and expert toxicology review provide structured evaluations, they remain partly subjective, and different raters, specialties, or scoring approaches might yield somewhat different quality and accuracy ratings. Fifth, while we examined like counts and common viewer response patterns, we did not systematically code all comments, and engagement metrics alone cannot show how viewers interpret or act on the content offline [6-9,11-13].

Finally, this cross‑sectional analysis from early 2025 cannot capture rapid shifts in platform algorithms, alcohol trends, or user behavior over time, underscoring the need for ongoing surveillance to track emerging hazardous consumption methods and evolving patterns of misinformation in digital environments [11-13].

## Conclusions

Hazardous alcohol consumption is prominently depicted in highly viewed YouTube videos, yet these videos rarely provide accurate risk information or explicit harm‑reduction messages. Many of the behaviors shown appear to be performed by legal‑age young adult men, and the same content is readily accessible to adolescents and younger viewers, raising concern that these portrayals may influence how youth perceive typical or desirable drinking. However, actual viewer demographics and causal effects could not be assessed in this study. 

Overall, video quality and reliability were low, and most scientific statements in informational videos contradicted established toxicology literature, suggesting that viewers may encounter persuasive but misleading narratives about alcohol. YouTube and similar platforms may represent important components of the alcohol‑related informational environment for the youth. Future work should evaluate whether clinician inquiry about online alcohol content exposure and platform‑based harm‑reduction or counter‑messaging strategies can effectively address misinformation and hazardous drinking norms.

## Appendices

Appendix A 

**Table 5:** YouTube search terms for hazardous alcohol consumption

alcohol inhalation	booty bumping alcohol	marijuana moonshine	player knockout drinking
inhaling alcohol vapor	rectal alcohol administration	weed alcohol shots	knockout drinking challenge
vaportini alcohol	vodka eyeballing	drunkorexia	rainbow drink challenge
smoking alcohol	eyeballing alcohol	anorexia and alcohol	drink the rainbow alcohol
alcohol vaporizer	alcohol in the eye	calorie saving binge drinking	drunken gummies
alcohol mist inhaler	drinking hand sanitizer	beer bong	alcohol gummies
powdered alcohol snorting	hand sanitizer shots	funneling alcohol	alcohol candy shots
snorting alcohol powder	drinking rubbing alcohol	alcohol funnel challenge	neknominate
alcohol powder challenge	alcohol-soaked tampons	borg drink	neck nomination drinking
alcohol enemas	tampon alcohol slimming	blackout Rage Gallon	first drink last drink challenge
butt chugging	vaginal alcohol use	borg drinking challenge	extreme binge drinking challenge
boofing alcohol	cannabis alcohol infusion	PK drinking game	

Appendix B 

**Table 6:** Global Quality Score (GQS) anchors and scoring Scoring: Each video receives a single GQS [15] from 1–5 based on the overall impression of quality, flow, and usefulness. Interpretation (for descriptive purposes): 4–5 = high quality; 3 = intermediate quality; 1–2 = low quality.

GQS score	Descriptor	Operational definition
1	Very poor quality	Disorganized or confusing video with little or no coherent educational value for viewers.
2	Poor quality	Some relevant information present, but major gaps, inaccuracies, or lack of clarity substantially limit usefulness.
3	Moderate quality	Generally understandable and somewhat helpful, but with notable omissions, limited depth, or minor inaccuracies.
4	Good quality	Clear, well‑structured, mostly accurate information that would be helpful for most lay viewers seeking guidance about the topic.
5	Excellent quality	Highly clear, well‑organized, comprehensive, and accurate content that would be very useful as an educational resource.

Appendix C 

**Table 7:** Modified DISCERN (mDISCERN) items and scoring Total score: Sum of item scores (range 0–5) [16,17]. Interpretation (for descriptive purposes): 4–5 = relatively high reliability; 2–3 = moderate reliability; 0–1 = very low reliability.

Item no.	mDISCERN item (video reliability criterion)	Scoring
1	The video clearly states its purpose and remains consistent with that purpose throughout.	1 = yes, 0 = no
2	The video draws on identifiable, credible sources (e.g., scientific references, clinician or toxicology expert) to support information.	1 = yes, 0 = no
3	The information is presented in a balanced way, without obvious promotional bias or one‑sided emphasis.	1 = yes, 0 = no
4	The video directs viewers to additional credible sources of information or further reading.	1 = yes, 0 = no
5	The video acknowledges uncertainty or limitations, such as gaps in evidence or areas where knowledge is still evolving.	1 = yes, 0 = no
